# Effects of Spatial Frequency Filtering Choices on the Perception of Filtered Images

**DOI:** 10.3390/vision4020029

**Published:** 2020-05-26

**Authors:** Sabrina Perfetto, John Wilder, Dirk B. Walther

**Affiliations:** 1 Human Biology Program, University of Toronto, Wetmore Hall, 300 Huron Street, Room 105, Toronto, ON M5S 3J6, Canada; sabrina.perfetto@mail.utoronto.ca; 2 Department of Psychology, University of Toronto, 100 St. George Street, Toronto, ON M5S 3G3, Canada; jdwilder@cs.toronto.edu; 3 Samsung Artificial Intelligence Center Toronto, 101 College Street, Suite 420, Toronto, ON M5G 1L7, Canada

**Keywords:** spatial frequencies, contrast normalization, Butterworth filter, natural scenes, scene gist, scene categorization

## Abstract

The early visual system is composed of spatial frequency-tuned channels that break an image into its individual frequency components. Therefore, researchers commonly filter images for spatial frequencies to arrive at conclusions about the differential importance of high versus and low spatial frequency image content. Here, we show how simple decisions about the filtering of the images, and how they are displayed on the screen, can result in drastically different behavioral outcomes. We show that jointly normalizing the contrast of the stimuli is critical in order to draw accurate conclusions about the influence of the different spatial frequencies, as images of the real world naturally have higher contrast energy at low than high spatial frequencies. Furthermore, the specific choice of filter shape can result in contradictory results about whether high or low spatial frequencies are more useful for understanding image content. Finally, we show that the manner in which the high spatial frequency content is displayed on the screen influences how recognizable an image is. Previous findings that make claims about the visual system’s use of certain spatial frequency bands should be revisited, especially if their methods sections do not make clear what filtering choices were made.

## 1. Introduction

Since the discovery of distinct spatial frequency channels in the human visual cortex [1], select spatial frequencies have been suspected to be involved in a variety of perceptual phenomena, including the processing of scene gist versus detailed scene information [2,3], in the processing of emotions in images of faces [4,5,6,7], as well as processing of objects in scene context [8]. Low and high spatial frequencies carry different aspects of the information contained in an image. Low spatial frequencies (LSF) concern global shape, whereas high spatial frequencies (HSF) carry edges, contours, and more detailed aspects.

Scene information that is gathered at a quick glance (the “gist” of a scene) is thought to rely predominantly on LSF in the scene, whereas perception of HSF subsequently provides information that feeds into recognition [2,8]. Evidence for the role of LSF versus HSF in scene perception from functional magnetic resonance imaging (fMRI) research is mixed. Several studies have shown higher activation of the scene-selective parahippocampal place area (PPA) for LSF than HSF [9,10], whereas other studies have shown strong activation of the PPA by HSF in both humans and monkeys [11,12] as well as more accurate decoding of scene content from the PPA for HSF than LSF scenes [13]. One study showed both effects within the same participants: higher activation of the PPA for LSF than HSF when filtered images were not normalized for contrast and higher activation for HSF than LSF when contrast was normalized [10].

In object recognition, HSF were thought to provide redundant information to the LSF, leading to the prediction that recognition performance would not be altered if HSF were removed [14,15]. However, the removal of either HSF or LSF had previously been shown to lead to slower reaction times, suggesting that both HSF and LSF are needed and are being used for object recognition [16]. In fact, the lack of a change in performance when removing HSF was later attributed to problems with the stimuli rather than observers not processing these frequencies. This conflicting information gives rise to the question of whether LSF really are dominating recognition and HSF are secondary, or if flaws with the filtering and presentation of the stimuli are confounding the results of these studies.

The processing of emotional facial expression under low or high pass filters has received much scrutiny. In a highly influential paper, the sensitivity of the amygdala to different frequencies was tested, showing higher activation of the amygdala for LSF than HSF face images [5]. This finding was supported by an EEG study [6] but challenged by another EEG study, which found no differences in the ERPs for LSF and HSF faces [7]. The difference between these studies may be due to differences in the contrast normalization procedure, either not normalizing contrast [5] (as far as is discernible from the methods section), or combining unnormalized frequency-filtered face images with complementary frequency-filtered textures [6], or normalizing for root-mean-square contrast [7].

Motivated by this set of contradictory results concerning the role of spatial frequencies across several fields of study, we here systematically explore the effect of three methodological choices when filtering images for spatial frequencies: contrast normalization (Experiment 1), filter shape (Experiment 2), and how to best display HSF images (Experiment 3). We use fast scene categorization as an experimental paradigm to test the effects of these choices on the perception of scene gist. We find that seemingly arbitrary decisions about the filtering procedure strongly affect perception, in some cases inverting the direction of effects. After characterizing these effects, we offer specific recommendations for filtering images for spatial frequencies. We make computer code available for spatial frequency filtering that follows these recommendations (https://osf.io/gphra/, 12 May 2020).

## 2. Experiment 1: Contrast Normalization

**Figure 1:**
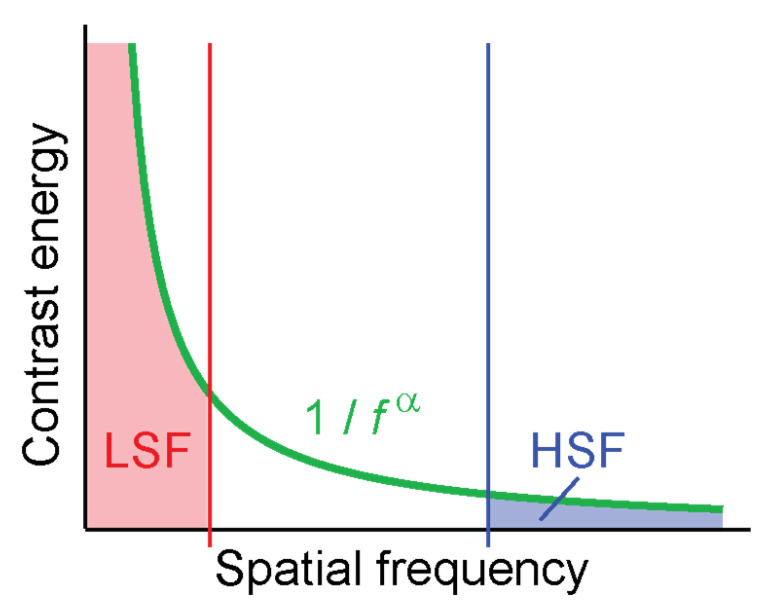
The $1/f^{\alpha }$ distribution of contrast in natural images leads to much higher overall contrast in LSF-filtered images (red) than HSF-filtered images (blue).

Contrast energy in natural images follows an inverse power law [17,18,19], with considerably more contrast energy contained in low than high spatial frequencies (Figure 1). When filtering images for high spatial frequencies, low spatial frequencies are suppressed or eliminated entirely (depending on the details of the filter, see Experiment 2). In typical natural images this means that a large part of the contrast energy (the part in the low spatial frequencies) is eliminated, leaving the image with overall less contrast energy than an image filtered for low spatial frequencies. When comparing human performance or neural signals in response to such filtered images, researchers might in fact simply compare performance for low-contrast images to performance for high-contrast images. Contrast is well known to directly drive activity of neurons in visual cortex [20,21,22,23].

A straightforward way to dissociate the effect of contrast energy from the effect of spatial frequencies is to normalize contrast in the filtered versions of the image. Contrast normalization can be achieved by first converting the pixel luminance values in all version of the image (unfiltered, HSF, LSF) to z-scores by subtracting the mean luminance and dividing by its standard deviation. Typically, luminance values for all three versions of the image are then jointly scaled to fit into the visible range of pixel values [0, 255]. This process ensures that all three versions of the image have the same mean luminance and the same root-mean-square (RMS) contrast (Appendix B of [24]). 

In this experiment, we compare scene categorization performance with and without contrast normalization. Participants see images of scenes from six scene categories in five different filtered conditions: Full spectrum (FS), contrast normalized; filtered for high spatial frequencies (HSF; >6 cpd) with contrast normalization; filtered for low spatial frequencies (LSF; <1 cpd) with contrast normalization; HSF without normalization; LSF without normalization.

### 2.1. Materials and Methods

#### 2.1.1. Participants

Twenty-one healthy young adults (18–28 years old; 15 female) participated in the experiment for partial course credit. All participants reported normal or corrected-to-normal vision and provided written informed consent. The experiment was approved by the Research Ethics Board of the University of Toronto (Protocol #30999) and followed the guidelines set out in the Declaration of Helsinki.

#### 2.1.2. Stimuli

We used a set of 432 color photographs (800 × 600 pixels) of scenes of six categories: beaches, forests, mountains, highways, city streets, and offices, downloaded from the internet. The images used in the experiments were rated as good exemplars of their categories in an online experiment [25]. The color photographs were converted to grayscale using Matlab’s rgb2gray function.

For spatial frequency filtering, we Fourier transformed each full-spectrum image $I_{\mathrm{FS}}$ using the two-dimensional Fast Fourier Transform implementation in Matlab, obtaining the amplitude spectrum *A* and the phase spectrum *φ*:

(1) $$\left(A,\varphi \right)=\mathrm{FFT}2\left(I_{\mathrm{FS}}\right)$$

We here did not include any measures to address artifacts arising from the periodicity assumption inherent in the Fast Fourier Transform implementation, although zero padding or mirroring the image at the edge are possible strategies to minimize such artifacts.

We multiplied the amplitude spectrum with a radially symmetric second-order Butterworth filter, which has been a reasonable and popular choice in other studies [2,7,13]. For a comparison of other filter choices see Experiment 2. We generated LSF images with a filter that retained spatial frequencies less than $\omega_{\mathrm{LSF}}=1$ cycle per degree (cpd), corresponding to 39 cycles per image (cpi), and HSF images with a filter that retained spatial frequencies greater than $\omega_{\mathrm{HSF}}=6$ cpd (232 cpi):

(2) $$\begin{array}{l} A_{\mathrm{LSF}}\left(f_{x},f_{y}\right)=A\left(f_{x},f_{y}\right)\cdot \frac{1}{1+{\left(\frac{f_{x}^{2}+f_{y}^{2}}{\omega_{\mathrm{LSF}}^{2}}\right)}^{n}}\\ A_{\mathrm{HSF}}\left(f_{x},f_{y}\right)=A\left(f_{x},f_{y}\right)\cdot \left[1-\frac{1}{1+{\left(\frac{f_{x}^{2}+f_{y}^{2}}{\omega_{\mathrm{HSF}}^{2}}\right)}^{n}}\right] \end{array}$$

Here, *n* = 2 is the order of the Butterworth filter. Note that the radial symmetry of the filter implies that cardinal and oblique orientations are filtered in the same way. Filtered amplitude spectra were recombined with the original phase spectrum and inverse Fourier transformed back into image space:

(3) $$\begin{array}{l} I_{\mathrm{LSF}}=\mathrm{IFFT}2\left(A_{\mathrm{LSF}}\cdot e^{i\varphi }\right)\\ I_{\mathrm{HSF}}=\mathrm{IFFT}2\left(A_{\mathrm{HSF}}\cdot e^{i\varphi }\right) \end{array}$$

For the “unnormalized” conditions we applied no further normalization to the LSF images. The mean (DC component) of the original images was passed to the LSF image by the low-pass filter. We explicitly added the mean of the original, full-spectrum image to the HSF image. Subsequently, we clamped any pixels with values less than zero to zero and pixels greater than 255 to 255.

For the contrast-normalized condition, we jointly normalized FS, HSF, and LSF versions of the same image as follows. We first converted the pixel values of each image separately to z-scores by subtracting the mean and dividing by the standard deviation:

(4) $$Z_{C}\left(x,y\right)=\frac{I_{C}\left(x,y\right)-\bar{I_{C}}}{\mathrm{STD}\left(I_{C}\right)}\forall C\in \left\{\mathrm{FS},\mathrm{HSF},\mathrm{LSF}\right\}$$

**Figure 2:**
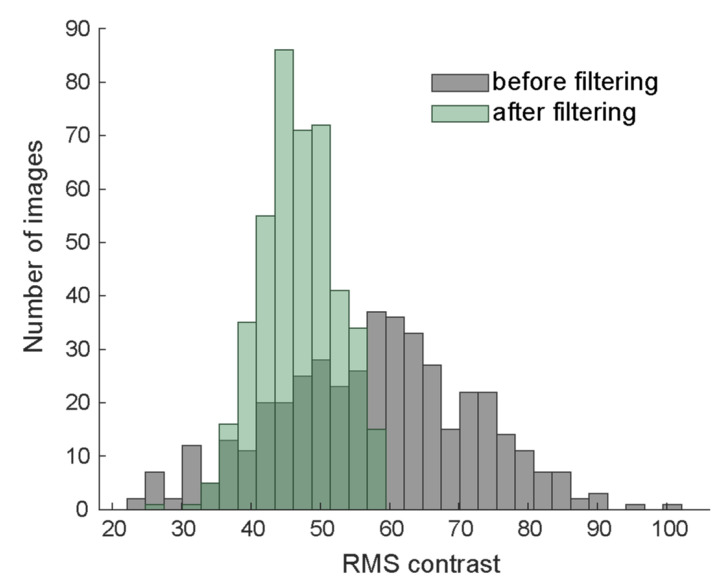
Distribution of root-mean-squared contrast before (gray) and after (green) filtering and contrast normalization.

This operation guarantees that all three versions of the same image have the same mean luminance and RMS contrast. The distribution of RMS contrast before and after filtering is shown in Figure 2. To limit values to a reasonable dynamic range for displaying, we clipped extreme values more than two standard deviation away from the mean to −2 and 2, respectively (on average 4% of pixels). The effect of clipping is visible on the high end of the distribution of RMS contrast (Figure 2). Since clipping slightly reduces contrast, we divided by the standard deviation once more to guarantee equal RMS contrast across all versions of the image. We then jointly scaled all three versions linearly to the visible range [0, 255] to arrive at the contrast-normalized images:

(5) $$\begin{array}{l} Z_{\min}=\underset{(x,y),C\in \left\{\mathrm{FS},\mathrm{HSF},\mathrm{LSF}\right\}}{\min}Z_{C}\left(x,y\right)\\ Z_{\max}=\underset{(x,y),C\in \left\{\mathrm{FS},\mathrm{HSF},\mathrm{LSF}\right\}}{\max}Z_{C}\left(x,y\right) \end{array}$$

(6) $$N_{C}\left(x,y\right)=\left\lfloor \frac{Z_{C}\left(x,y\right)-Z_{\min}}{Z_{\max}-Z_{\min}}\cdot 255\right\rfloor \forall C\in \left\{\mathrm{FS},\mathrm{HSF},\mathrm{LSF}\right\}$$

**Figure 3:**
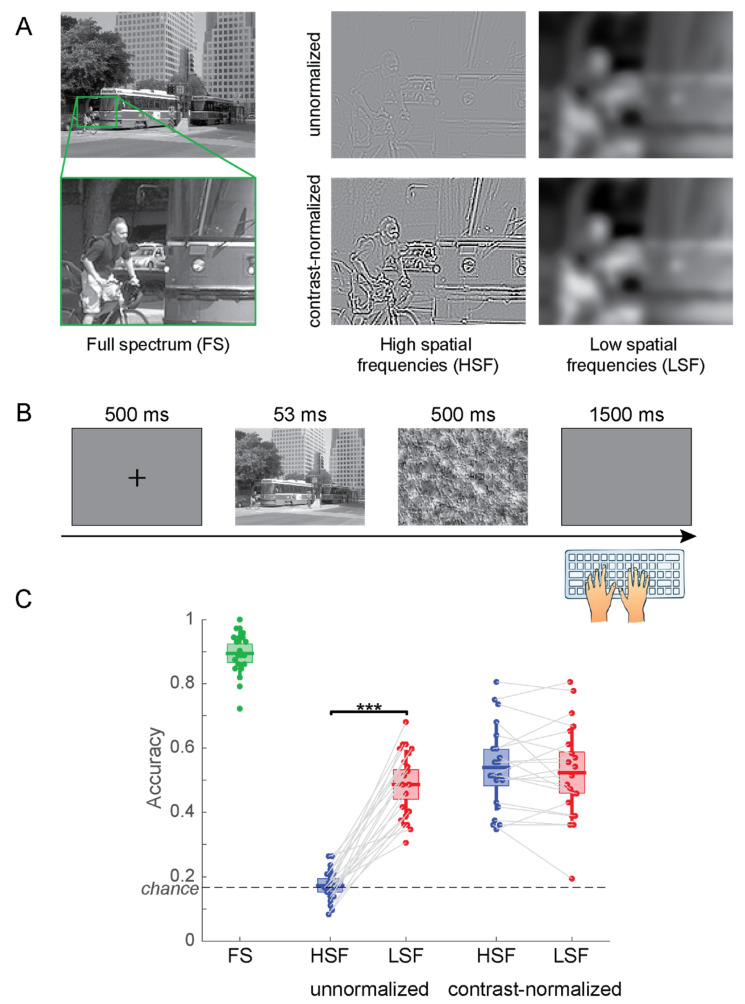
(**A**) Example stimuli for Experiment 1. To better illustrate the effects of the filtering choices, we display enlarged sections of the actual stimuli. (**B**) Time course of the experiment. (**C**) Accuracy of scene categorization for the five types of stimuli. The center lines of the boxes indicate the mean, the extent of the box the standard error of the mean, and the whiskers the standard deviation of the individual data points. *** *p* < 0.001.

Experiment 1 included five conditions: unnormalized HSF $\left(I_{\mathrm{HSF}}\right)$, unnormalized LSF $\left(I_{\mathrm{LSF}}\right)$, contrast-normalized full spectrum $\left(N_{\mathrm{FS}}\right)$, contrast-normalized HSF $\left(N_{\mathrm{HSF}}\right)$, and contrast-normalized LSF $\left(N_{\mathrm{LSF}}\right)$. See Figure 3A for example stimuli.

#### 2.1.3. Apparatus

The experiment was performed in a dark experiment room. All stimuli were presented full screen on a cathode ray tube monitor (Dell) at a resolution of 800 × 600 pixels and a refresh rate of 150 Hz. Note that we did not attempt to linearize the luminance response of the monitor, since this is not commonly done when displaying photographic images. Participants were seated 57 cm from the screen, with their chin and forehead supported by a chin rest. The experiment was controlled with a personal computer running the Windows 7 operating system and custom Matlab code using the Psychophysics Toolbox [26].

#### 2.1.4. Procedure

The experiment consisted of three phases: training, linear ramping and testing. For each participant, 72 images (12 from each category) were chosen randomly and used repeatedly for practice and ramping. Three hundred and sixty images were reserved for testing and shown only once.

In the practice phase, participants saw full-spectrum images $\left(N_{\mathrm{FS}}\right)$ for 200 ms, immediately followed by a perceptual mask for 500 ms. Perceptual masks were constructed from noise textures with a broad spatial frequency spectrum. Note that frequency-matched masks would increase task difficulty while at the same time isolate stimulus-enabled performance. We here opted for broadband masks to keep them the same for all experimental conditions. A blank screen prompted participants to respond with the category of the presented image (beach, forest, mountain, highway, city or office) by pressing one of six keys on a standard computer keyboard: S, D, F for the left hand, and J, K, L for the right hand (Figure 3B). The assignment of keys to categories was randomized for each participant. Since memory was not being tested during the experiment, each participant was allowed to write their key assignment on a small piece of paper, and reference it to learn the keys. Participants were asked to respond as accurately as possible and not pay attention to their response time. Participants heard a short beep through headphones when they made an error. Once participants achieved an accuracy of 90% they proceeded to the ramping phase.

The ramping phase used the same images as the practice phase and steadily ramped down the stimulus onset asynchrony (SOA) from 200 to 33 ms over 54 trails to prepare participants for the following testing phase. The testing phase consisted of 360 trials with a fixed SOA of 53 ms (eight frames). Images were counterbalanced for the six scene categories and the five image conditions (full spectrum, HSF unnormalized, LSF unnormalized, HSF contrast normalized, LSF contrast normalized), with a total of 12 trials for each combination of category and condition. Participants saw each of the 360 test images exactly once. They no longer received any feedback for incorrect trials. Accuracy of responses was tallied separately for each of the five conditions.

### 2.2. Results

The participants categorized FS images correctly in 89.4% of the trials. When contrast was not normalized, the categorization accuracy was 17.3% for HSF and 48.6% for LSF images (Figure 3C). With contrast normalized across filtered images, participants categorized HSF images correctly at 53.9% and LSF images at 52.3%. To evaluate the effects of contrast normalization and frequency filtering separately, we performed a 2 (HSF vs. LSF) × 2 (no normalization vs. contrast normalization) fixed-effects Analysis of Variance (ANOVA). The ANOVA showed significant main effects for spatial frequency (*F*(1,80) = 34.25; *p* = 1.00 × 10^−7^) and normalization (*F*(1,80) = 63.08; *p* = 1.05 × 10^−11^) as well as their interaction (*F*(1,80) = 42.01; *p* = 6.89 × 10^−9^). Planned paired t tests show that the difference in accuracy between HSF and LSF images is highly significant without contrast normalization (*t*(20) = −13.11; *p* = 2.79 × 10^−11^) but not when normalizing the filtered images for RMS contrast (*t*(20) = 0.90; *p* = 0.378).

These results are clearly driven by image contrast alone; HSF images without contrast normalization have a much lower contrast than any of the other experimental conditions. Hence, the decision whether to normalize contrast in filtered images pre-determines the outcome of the experiment. When contrast is not normalized, HSF images are guaranteed to be perceived significantly worse, simply because they contain less contrast energy. We therefore argue that an experiment comparing perception of HSF and LSF images without contrast normalization primarily evaluates the effect of image contrast rather than spatial frequencies on perception.

## 3. Experiment 2: Filter Shape

**Figure 4:**
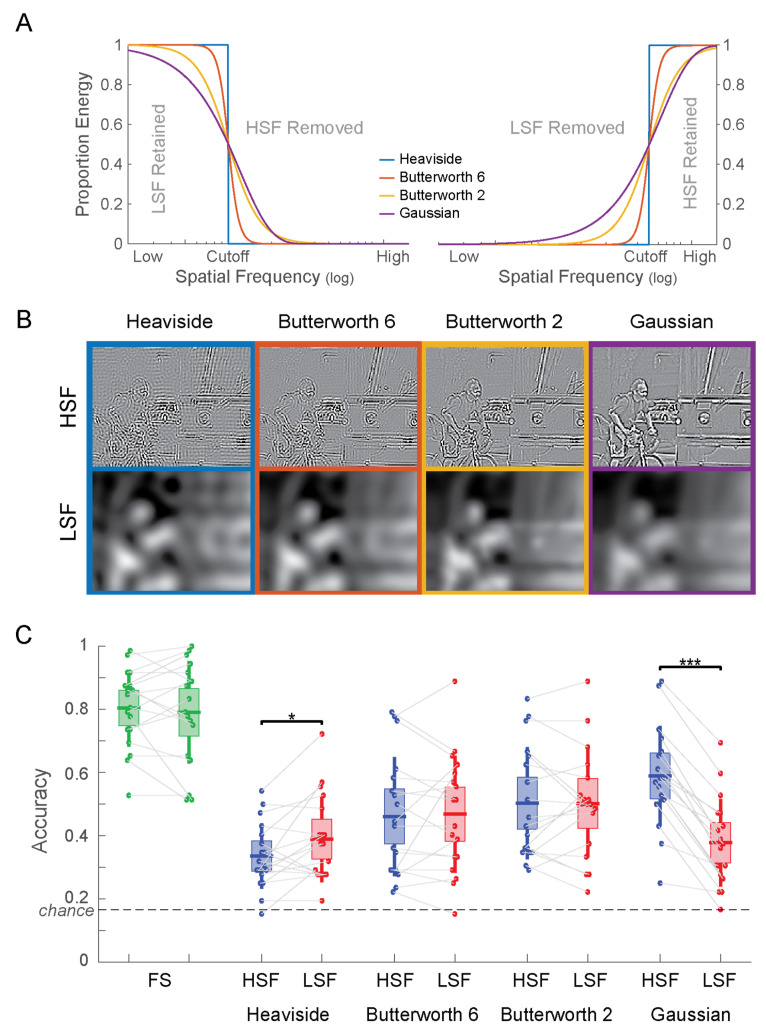
(**A**) Frequency response of the four filter types both as low-pass filters (left) and as high-pass filters (right). (**B**) Example stimuli for Experiment 2. Note the strong ringing artifacts for the Heaviside filter, which successively decrease for smoother filters (from left to right). In the Gaussian HSF image (top-right) spillover of LSF into the HSF are clearly visible, e.g., on the face and the arms of the cyclist. (**C**) Accuracy of scene categorization for Experiment 2. * *p* < 0.05; *** *p* < 0.001.

Another important determinant for filtering images for spatial frequencies is the shape of the frequency filter. We here consider four choices for filters in the frequency domain (Figure 4A).

A Heaviside filter consists of a sharp cutoff of spatial frequencies using a step function. This filter leads to the cleanest separation of spatial frequencies. However, due to its sharpness in the frequency domain, the filter generates a broad spectrum of ringing artifacts in the image domain (Figure 4B, left column).

This issue is mitigated by using a smoother, more gradual transition between frequencies. A common choice for a smooth frequency filter is a Gaussian function. Filtering with a Gaussian filter can either be implemented as a multiplication of the amplitude spectrum with a Gaussian filter or by convolving the image with a Gaussian kernel (since the Fourier transform of a Gaussian is a Gaussian). The latter method is particularly common, since it is implemented as a “Gaussian blur filter” in common image editing software products. A Gaussian filter does indeed result in a drastic reduction of ringing artifacts (Figure 4B, right column), albeit at the expense of a precise cutoff in spatial frequency. In fact, since a Gaussian function has infinite support, the undesired part of the frequency spectrum is never entirely excluded. That is, a Gaussian high-pass filter will generally contain at least some amount of low spatial frequencies in the image. In practice, this is usually mitigated by truncating filters to a fixed size or simply by the limits of machine precision. However, this is not a clean way of determining frequency cutoffs. The issue of unclean filtering can be seen distinctly in the Gaussian HSF image (Figure 4B, top-right), which clearly has spillover from the LSF.

First developed for the filtering of electrical signals, Butterworth filters were designed as a compromise between sharpness of the frequency cutoff and the suppression of filtering artifacts [27]. The functional form of the Butterworth filter (Equation (2)) includes an exponent n, the “order” of the Butterworth filter. The order trades off artifact suppression with the sharpness of the filter. We here consider Butterworth filters of orders 2 and 6, which fall between the extremes of a Heaviside filter and a Gaussian filter in their frequency response properties (Figure 4A).

Note that it is only possible to properly evaluate the effect of the filters on the perception of filtered images when normalizing contrast between HSF and LSF. Otherwise, LSF images would be much easier to recognize than HSF images irrespective of the choice of the filter because they would have higher contrast (see Experiment 1).

### 3.1. Materials and Methods

#### 3.1.1. Participants

Twenty-one healthy young adults (18–21 years old; 16 female) participated in the experiment for partial course credit. All participants reported normal or corrected-to-normal vision and provided written informed consent. The experiment was approved by the Research Ethics Board of the University of Toronto (Protocol #30999) and followed the guidelines set out in the Declaration of Helsinki. The data of three female subjects were excluded from the analysis due to poor performance for FS images (less than 50% correct).

#### 3.1.2. Stimuli

We used the same set of photographs of natural scenes as in Experiment 1. Images were Fourier-transformed, and the amplitude spectra were filtered for HSF (>6 cpd) and LSF (<1 cpd) as follows:

Heaviside filter:

(7) $$\begin{array}{l} A_{\mathrm{LSF}}\left(f_{x},f_{y}\right)=\{\begin{array}{cc} A\left(f_{x},f_{y}\right) & \left(f_{x}^{2}+f_{y}^{2}\right)<\omega_{\mathrm{LSF}}^{2}\\ 0 & \text{otherwise} \end{array}\\ A_{\mathrm{HSF}}\left(f_{x},f_{y}\right)=\{\begin{array}{cc} A\left(f_{x},f_{y}\right) & \left(f_{x}^{2}+f_{y}^{2}\right)>\omega_{\mathrm{HSF}}^{2}\\ 0 & \text{otherwise} \end{array} \end{array}$$

Butterworth filters were applied to the amplitude spectrum following Equation (2), with *n* = 2 and *n* = 6, respectively. We performed Gaussian filtering in the image domain by convolving the image with a two-dimensional Gaussian kernel to replicate the common practice of applying the Gaussian blur to the image directly:

(8) $$\begin{array}{l} I_{\mathrm{LSF},\mathrm{Gaussian}}=I*G\left(0,\sigma_{\mathrm{LSF}}\right)\\ I_{\mathrm{HSF},\mathrm{Gaussian}}=I-I*G\left(0,\sigma_{\mathrm{HSF}}\right) \end{array},$$

where $\sigma_{\mathrm{LSF}}$ and $\sigma_{\mathrm{HSF}}$ were chosen such that the corresponding Gaussian kernel in frequency space has its half maximum at the cutoff frequencies $\omega_{\mathrm{LSF}}$ and $\omega_{\mathrm{HSF}}$, respectively:

(9) $$\sigma =\frac{f_{s}}{\omega \cdot \pi \cdot \sqrt{2\cdot \log2}}$$

Here, $f_{s}=20.7$ pixels/degree is the sampling frequency.

The corresponding HSF and LSF images for each filter were jointly contrast-normalized as described in Experiment 1. Examples of filtered images are shown in Figure 4B. Notice that both LSF and HSF images filtered with the Heaviside filter show perceptible ringing artifacts (ripples across the image). These artifacts are successively diminished in the two versions of the Butterworth filter and not visible in the images filtered with the Gaussian filters. This reduction in ringing artifacts comes at the cost of the sharpness in the frequency cutoff (Figure 4A).

#### 3.1.3. Procedure

The same hardware and viewing distances as in Experiment 1 were used in this experiment. Due to the large number of conditions, each participant performed two versions of the scene categorization experiment back-to-back. Version A included FS as well as HSF and LSF images filtered with Heaviside and with Gaussian filters. Version B included FS as well as HSF and LSF images filtered with Butterworth filters of orders 2 and 6.

Participants performed practice and ramping trials as described in Experiment 1, followed by the test trials for Version A (or B), and then test trials for Version B (or A), all with the same assignment of keys to categories. The order of the two versions was randomly assigned to each participant. As a consequence, participants viewed each test image twice, once in each of the two versions, albeit generally in differently filtered conditions.

### 3.2. Results

Categorization accuracy for all experimental conditions is shown in Figure 4C. Performance for the FS images was the same in versions A (80.4%) and B (79.1%; *t*(17) = 0.577; *p* = 0.57). A 2 (HSF vs. LSF) × 4 (filter shape) ANOVA revealed a significant main effect for filter shape (*F*(3,136) = 5.52; *p* = 0.0013) but not for spatial frequency (*F*(1,136) = 2.00; *p* = 0.16). The interaction between the factors was significant (*F*(3,136) = 4.95; *p* = 0.0027). Planned paired t tests show significant differences in the accuracy for HSF vs. LSF images for the Heaviside filter (*t*(17) = −2.373; *p* = 0.030) and the Gaussian filter (*t*(17) = 9.971; *p* = 1.61 × 10^−8^) but not for the 6th order Butterworth filter (*t*(17) = −0.297; *p* = 0.77) or the second order Butterworth filter (*t*(17) = 0.034; *p* = 0.97).

Remarkably, the direction of the difference switches from the Heaviside filter (HSF < LSF) to the Gaussian filter (HSF > LSF). The latter result is presumably due the spillover of LSF image content into the Gaussian HSF image (Figure 4B, top right). These results powerfully demonstrate that the particular choice of the frequency filter can pre-determine the result of comparisons between high and low spatial frequencies.

## 4. Experiment 3: Displaying HSF Images

Whereas the first two experiments addressed issues with the filtering procedures, this experiment addresses choices in how to display HSF images to participants, which nevertheless influence participants’ performance.

When encoding images in digital computers, we follow the arbitrary convention that dark pixels have small values and bright pixels large values assigned to them. The opposite assignment would be just as valid as long as it is followed consistently—after all, dark pixels require more ink to be printed than bright pixels. The polarity of this convention does not matter for any of the filtering choices discussed so far. But it becomes important for the decision how to display HSF images.

HSF images prior to any normalization steps usually contain positive as well as negative pixel values. The same is not true for LSF images, because LSF images include the mean luminance of the image (the “DC component”) at the very low end of the frequency spectrum. When normalizing HSF images to the visible range of pixels values (whether jointly contrast normalized or not), much of the image typically assumes a medium gray level (corresponding to zero in the filter output) with both dark (negative) and bright (positive) lines. The sign largely depends on the contrast polarity of local edges (their phase in Fourier space). Specifically, finite-width contour lines in the original image will frequently result in pairs of bright and dark lines in the HSF image. As a result, HSF images often look alien and unfamiliar, similar to photographic negatives. When choosing to display HSF images in this fashion, the task (e.g., scene categorization) is made more difficult than necessary, because observers are less fluent in processing images encoded this way.

The HSF content of images is typically related to sharp edges and contours. People are quite sensitive to such contour information. In fact, many visual representations generated by humans rely exclusively on marking contours—from prehistoric cave drawings to the drawings by Picasso, from the first squiggly drawings of preschoolers to technical drawings of complex machines. The majority of these visual representations are produced by making dark markings on a bright background—charcoal on rock wall, pencil on paper, ink on canvas. Hence, observers are much more used to contour information being shown as dark lines on a bright background rather than dark and bright markings on a medium-gray background.

To explore the effect of this cultural habituation, we here map both negative and positive values in HSF images to dark tones on a bright background. As a result, HSF images resemble detailed line drawings, which are more familiar to observers than HSF images with dark and bright lines on a gray background. This operation is similar in spirit to the MIRAGE model of edge representations [28], but for fully two-dimensional, complex, real-world images instead of one-dimensional luminance patterns.

### 4.1. Materials and Methods

#### 4.1.1. Participants

Twenty-two healthy young adults (18–22 years old; 17 female) participated in the experiment for partial course credit. All participants reported normal or corrected-to-normal vision and provided written informed consent. The experiment was approved by the Research Ethics Board of the University of Toronto (Protocol #30999) and followed the guidelines set out in the Declaration of Helsinki. The data of one male subject were excluded from the analysis due to poor performance for FS images (less than 50% correct).

#### 4.1.2. Stimuli

We used the same image set as in Experiment 1, filtered for HSF and LSF using a second-order Butterworth filter as described in Equation (2). In addition, we generated a version of the HSF, in which both negative and positive values were mapped onto dark shades according to their absolute numerical values, whereas values close to zero were mapped into light shades. This was achieved by computing the negative absolute value of the filtered image immediately following the inverse Fourier transform (Equation (3)):

(10) $$I_{\mathrm{HSF},\mathrm{dark}}=-\left|H_{\mathrm{HSF}}\right|$$

**Figure 5:**
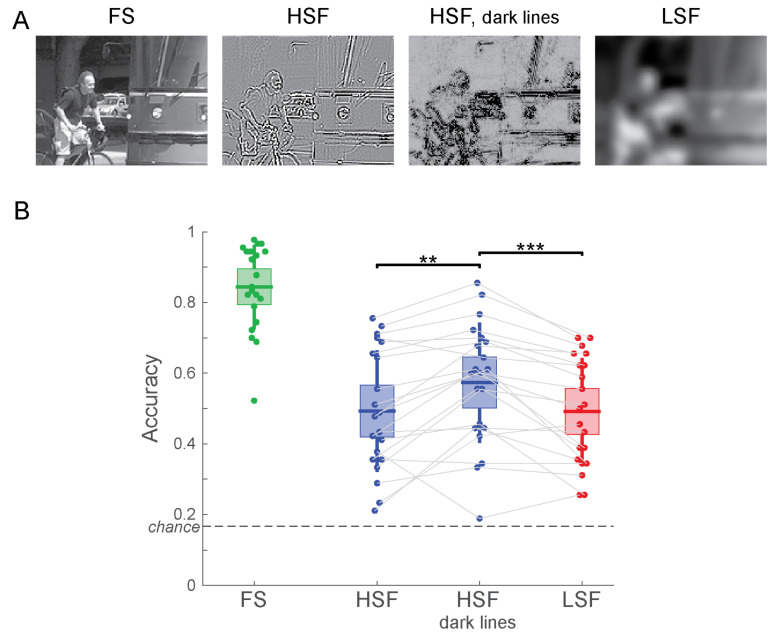
(**A**) Example stimuli for Experiment 3. (**B**) Accuracy of scene categorization for Experiment 3. ** *p* < 0.01; *** *p* < 0.001.

Following this operation, all four versions of the image (FS, LSF, HSF, HSF with dark lines) were jointly contrast normalized as described in Equations (5) and (6). Example stimuli are shown in Figure 5A.

#### 4.1.3. Procedure

The same setup and procedures were used as in Experiment 1. Instead of the five stimulus conditions in Experiment 1, we here only had four conditions: FS, LSF, HSF, and HSF with dark lines. Consequently, there were 15 test trials per condition and category for each participant.

### 4.2. Results

Participants categorized the FS image with 84.4% accuracy, replicating the FS results from the previous two experiments. The accuracy for HSF images was 49.3%, which significantly increased to 57.4% when mapping negative and positive values to dark tones (*t*(20) = −3.62; *p* = 0.0017; Figure 5B). For LSF images, the accuracy was at 49.2%, and it did not differ from the accuracy for HSF images (*t*(20) = 0.0439; *p* = 0.97). This result replicates the lack of a difference in categorization performance between contrast-normalized HSF and LSF images found in Experiment 1. When mapping HSF values to dark tones, on the other hand, scene categorization was significantly more accurate for HSF than for LSF images (*t*(20) = 4.44; *p* = 0.00025).

These results demonstrate that human observers are perceptually more fluent with images that show sharp contour information as dark markings on a bright background rather than as bright and dark markings on a gray background. Note that this choice in displaying HSF information does not change the spatial frequency-specific content or the RMS contrast of the filtered image: all filtering operations were performed exactly the same, and contrast was normalized jointly for all four versions of the image. Merely the way the filtered images were displayed was altered.

## 5. Discussion

In the three experiments of this study, we have demonstrated that simple choices in filtering procedures and in displaying the results of spatial frequency filtering can completely determine the effect of high and low spatial frequency content on the perception of briefly presented scenes. The first experiment highlights the importance of contrast normalization when comparing spatial frequency content of images. Low spatial frequencies naturally contain more contrast than high spatial frequencies [17,18,19]. Neurons in early visual cortex are sensitive to both spatial frequency and contrast [1]. When attempting to isolate the role of spatial frequencies from that of contrast in perception, normalization for equal contrast is critical [29]. When measuring contrast sensitivity functions, for instance, equalizing for contrast between gratings of different spatial frequencies is a matter of course [1]. Unfortunately, this is not the case for many studies of the role of spatial frequencies in scene perception, including recent studies [30,31]. Not normalizing contrast leaves HSF stimuli at a visible disadvantage and does not isolate the spatial frequency variable. When normalizing for contrast, accuracy of scene categorization for HSF images increased from chance level to just as high as performance for LSF images (Figure 3C).

Whether LSF and HSF are normalized for equal contrast is a likely explanation for fMRI results of scene perception as well. Kauffmann and colleagues found stronger activation of the parahippocampal place area by LSF compared to HSF scenes when they were not normalized. Within the same experiment, the direction of the effect reversed when contrast was normalized [10]. Moreover, a study employing multi-voxel pattern analysis found that scene categories could be decoded significantly *better* from brain activity elicited by HSF than LSF scenes that were normalized for equal contrast [13].

One could make the argument that filtering scenes without normalizing contrast preserves the natural distribution of contrast energy over spatial frequencies. In this case, care should be taken to qualify any differences in the effect of spatial frequencies as most likely being related to contrast.

The specific shape of the frequency filter also affects perception. When comparing the images generated with different filters, a clear tradeoff between artifacts in the output image and frequency content available in the image emerged. The sharper the cutoff is between the low and high pass sets, the more ringing artifacts are visible in the filtered images (Figure 4B). To mitigate these artifacts, a filter with a smoother transition should be used. A Gaussian filter fits this criterion, albeit at the loss of a well-defined frequency cutoff due to the infinite support of the Gaussian function. Constructed specifically for a flat frequency response in the pass region, Butterworth filters trade off the clarity of the frequency separation with suppressing ringing artifacts. We here tested Butterworth filters of orders 2 and 6. The choice of the filter shape clearly affects human perception. We found significantly better scene categorization performance for LSF than HSF for the Heaviside filter, with the opposite pattern for the Gaussian filter. The two Butterworth filters did not show any clear advantage for HSF or LSF (Figure 4C).

While people might see blurry LSF images in real-life situations (forgetting to put on one’s spectacles, views through frosted glass, a camera with unfocussed optics), HSF images with their mix of black and white lines on a grey background look unnatural to human observers. In Experiment 3, we addressed this issue using a simple manipulation—we displayed pixels with large HSF values as dark pixels, irrespective of their sign. This choice makes HSF images appear more like drawings of dark contours on a bright background—something that is familiar to human observers. This manipulation boosted accuracy of categorizing HSF images so that they were now categorized more accurately than LSF images (Figure 5B).

In all, our experiments highlight that the effect of spatial frequencies on human perception can be entirely driven by specific choices of the filter shape, the way filtered images are normalized, and the way HSF images are displayed. Note that we have not even manipulated the most obvious variable—the frequency cutoff. All of our experiments used the same cutoffs (<1 cpd for LSF, >6 cpd for HSF). When targeting specific frequency channels, e.g., in striate cortex, it may be advisable to use bandpass filters instead of highpass or lowpass filters. Obviously, these choices will have a large influence on human perception of the filtered images.

Our work provides specific recommendations for research practices. First and foremost, the details of the frequency filtering must be clearly reported in the methods section. Ideally, filtering code and filtered stimuli should be made available alongside published manuscripts. We make all of our stimuli, the filtering code, the raw data from the experiments, and the analysis code available at (https://osf.io/gphra/, 12 May 2020).

Based on our results, we offer clear recommendations for filtering procedures to ensure the fair display of HSF and LSF frequency bands. Contrast normalization allows for the spatial frequency variable to be isolated from the effects of contrast energy. Therefore, **contrast normalization is essential** for isolating the role of spatial frequencies from that of image contrast. Controlling the contrast of stimuli is a matter of course in virtually all of visual psychophysics. Spatial frequency filtering of images should not be an exception to this rule. When experiments do not normalize contrasts, reasons should be provided, and the potentially confounding effect of contrast should be acknowledged. Choosing the shape of the frequency filter is a balance between the sharpness of the filter and the suppression of ringing artifacts. We found the **second order Butterworth** filter to be a good compromise. Butterworth filters are smooth around the cutoff frequency and flat in the pass band, thus avoiding confusing artifacts. However, they are not so smooth that they blend the frequency content between the LSF and HSF versions, as Gaussian filters inevitably do. Lastly, we cautiously recommend **displaying large HSF value of either sign as dark pixels** on a bright background, as this allows observers to process the images more fluently. This last recommendation may be somewhat controversial. By computing the negative absolute value of HSF-filtered pixels, it introduces a non-linear operation and thereby potentially new low spatial frequency components. We view those operations as an alternate way of displaying images whose original LSF content has already been removed by the high-pass filter. Therefore, the remaining image content is related only to the HSF content of the original image.

Interestingly, following our recommendations turns a clear superiority of LSF images (Exp. 1, unnormalized) into a clear superiority of HSF images (Exp. 3, black lines). This finding supports the notion that detailed structural information rather than a blurry LSF version of the image underlies perception of scene gist. This view underscores the importance of contours and their junctions for scene perception [32,33,34,35] as well as navigation [36].

In light of the findings of this paper, effects of spatial frequencies in scene, object and face perception should be revisited and scrutinized for specific choices in generating the filtered stimuli. If results are in doubt, registered replication attempts might offer an avenue to reconcile disparate results with clearly defined filtering strategies.

The role of spatial frequencies in visual perception may only be a small piece of the much larger puzzle that the brain has to solve to affect the seemingly effortless feat of seeing our complex visual environment. However, it is an important piece, whose methodologically sound understanding will bring us closer to understanding the full picture of biological vision.
